# MECHANISMS OF DEVELOPMENT AND REGENERATION IN HYDRA

**DOI:** 10.1093/geroni/igac059.1068

**Published:** 2022-12-20

**Authors:** Celina Juliano, Jack Cazet, Abby Primack

**Affiliations:** University of California, Davis, Davis, California, United States; University of California, Davis, Davis, California, United States; University of California, Davis, Davis, California, United States

## Abstract

Hydra vulgaris is a small and simple aquatic animal capable of whole-body regeneration and has negligible senescence. The entire animal, including the nervous system, is composed of about 25 cell types, and can regenerate from a fragment of tissue as small as ~300 cells. In addition, all cell types are continually renewed in the uninjured adult as part of normal homeostasis; every differentiated cell type is replaced approximately every 20 days, which likely contributes to its lack of aging. The remarkable features of Hydra are enabled by three distinct populations of stem cells that support the three lineages that make up the adult Hydra – the ectodermal epithelial lineage, the endodermal epithelial lineage, and the interstitial lineage (includes the neurons). A major goal of our laboratory is to understand the gene regulatory networks that control the specification of all Hydra cell types in the uninjured (homeostatic) state and then understand how injury triggers these differentiation pathways at unexpected locations during regeneration. Using high throughput genomics approaches such as scRNA-seq, ATAC-seq, and Cut&Tag, we have transcriptionally defined every cell type in Hydra and identified putative transcriptional regulators for each cell type. This includes the 11 neuronal subtypes that comprise the nerve net that spans the entire length of the Hydra body. We are currently leveraging these data to conduct functional testing of key putative regulators and to identify injury inputs into cell specification events during regeneration.

